# Commentary on: Artificial Intelligence in Surgical Evaluation: A Study of Facial Rejuvenation Techniques

**DOI:** 10.1093/asjof/ojad049

**Published:** 2023-06-14

**Authors:** Al Aly, Y Edward Wen, Cyrus Steppe

See the Original Article here.

In this Video Commentary, we agree with the authors’ desire to quantify the improvement in “emotion” of varying facelift techniques through artificial intelligence (AI), as a “proof of concept” approach to objectively judging surgical results (Video).^1^ We mildly disagree with the authors in using “emotion” as a way of judging results and felt that a more practical and clinically relevant measure would be determining the effects on attractiveness of these varying techniques. We also prefer to use “crowdsourcing” over AI to investigate the type of questions asked in this paper because it can give the researcher the opinion of a very large number of evaluators, like AI, but with the benefit of allowing the gathering of information on the demographics of the evaluators, leading to a much clearer picture of how different groups judge the level of attractiveness.

## Supplementary Material

ojad049_Supplementary_Data
